# Transcriptomic analysis of the maize inbred line Chang7-2 and a large-grain mutant *tc19*

**DOI:** 10.1186/s12864-021-08230-9

**Published:** 2022-01-04

**Authors:** Yanrong Zhang, Fuchao Jiao, Jun Li, Yuhe Pei, Meiai Zhao, Xiyun Song, Xinmei Guo

**Affiliations:** 1 College of Agronomy, Qingdao Agricultural University, Qingdao, 266109 Shandong China; 2 Key Laboratory of Major Crop Germplasm Innovation and Application in Qingdao, Qingdao, 266109 Shandong China; 3 College of Life Science, Qingdao Agricultural University, Qingdao, 266109 Shandong China

**Keywords:** Maize, Grain size, Mutant, Hormone, Transcriptome

## Abstract

**Backgrounds:**

Grain size is a key factor in crop yield that gradually develops after pollination. However, few studies have reported gene expression patterns in maize grain development using large-grain mutants. To investigate the developmental mechanisms of grain size, we analyzed a large-grain mutant, named *tc19*, at the morphological and transcriptome level at five stages corresponding to days after pollination (DAP).

**Results:**

After maturation, the grain length, width, and thickness in *tc19* were greater than that in Chang7-2 (control) and increased by 3.57, 8.80, and 3.88%, respectively. Further analysis showed that grain width and 100-kernel weight in *tc19* was lower than in Chang7-2 at 14 and 21 DAP, but greater than that in Chang7-2 at 28 DAP, indicating that 21 to 28 DAP was the critical stage for kernel width and weight development. For all five stages, the concentrations of auxin and brassinosteroids were significantly higher in *tc19* than in Chang7-2. Gibberellin was higher at 7, 14, and 21 DAP, and cytokinin was higher at 21 and 35 DAP, in *tc19* than in Chang7-2. Through transcriptome analysis at 14, 21, and 28 DAP, we identified 2987, 2647 and 3209 differentially expressed genes (DEGs) between *tc19* and Chang7-2. By using KEGG analysis, 556, 500 and 633 DEGs at 14, 21 and 28 DAP were pathway annotated, respectively, 77 of them are related to plant hormone signal transduction pathway. *ARF3*, *AO2*, *DWF4* and *XTH* are higher expressed in *tc19* than that in Chang7-2.

**Conclusions:**

We found some DEGs in maize grain development by using Chang7-2 and a large-grain mutant *tc19*. These DEGs have potential application value in improving maize performance.

**Supplementary Information:**

The online version contains supplementary material available at 10.1186/s12864-021-08230-9.

## Background

Maize is an important human food, livestock feed, and bioenergy crop of great economic significance. Global maize production reached 1.1 billion tons in 2019 according to the Food and Agriculture Organization, providing a significant amount of food, feed, and bioenergy raw materials. The number of ears per unit area, number of grains per ear, and grain weight are the three main factors of maize yield [1]. Among these, grain weight is the primary factor affecting yield, because decreased grain weight cannot be compensated for by other yield factors. Grain size and filling degree are the main factors that affect grain weight, and thus grain size is an important trait affecting grain weight.

Maize seeds are composed of an embryo, endosperm, and seed coat. The maize embryo includes the germ, germ sheath, hypocotyl, radicle, sheath, and shield. The maize endosperm accounts for more than 80% of the volume and dry weight of the whole seed and is the most important component of maize seeds. The weight and quality of maize seeds are determined by the development, proliferation, and enrichment of endosperm cells. The maize endosperm is developed by the fusion of a male gamete with two polar nuclei. Maize endosperm development includes several stages: primary endosperm nuclear division, syncyte stage, syncyte cytochemistry, mitotic boom stage, nutrient storage stage, and dehydration and maturation stage [2].

Some genes associated with maize grain development have been identified using mutants. *Opaque2* encodes endosperm specific transcription factor. *Opaque2* functions in the expression of 22 KDa zeins [3]. *Shrunken 1* has a role in sucrose synthetase, the starch content in shrunken1 mutant endosperm is less than wildtype [4]. In addition, tens of genes in responding for maize defective kernel mutants have been cloned. *Dek2* encodes a pentatricopeptide repeat protein which functions in nad1 mRNA splicing [5]. *Dek15* affects kernel development by encoding the cohesion-loading complex subunit SCC4 [6]. *Dek35* encodes a PPR protein that affects cis-splicing of mitochondrial nad4 intron1 [7]. D*ek44* encodes mitochondrial ribosomal protein L9 [8]. Embryo defective 14 encodes a plastid-targeted cGTPase essential for embryogenesis [9]. Recently, one study found that the maize *Big Grain 1 Homolog 1* (*ZM-BG1H1*) overexpression is associated with increased ear kernel row number and total ear kernel number and mass [10]. In the case of maize grain development, most studies focused on small-grain mutants, only a few studies used large-grain mutants.

Grain development is a complex process regulated by plant hormones [11]. Genes associated with auxin, brassinolide, cytokinin, abscisic acid, and gibberellin are crucial for grain size. At present, many plant hormone–related genes have been identified to play critical roles in grain development. Maize *ARGOS8* negatively regulates ethylene responses. Overexpressing *ARGOS8* reduced ethylene sensitivity and improved grain yield under drought stress conditions [12]. The transcription factor *basic region/leucine zipper motif 53* (*bZIP53*) expression significantly promoted the expression of *cellulose synthase gene 1* (*CesA1*) which is involved in kernel development regulated by gibberellin [13]. *NEEDLE1* encodes an ATP-dependent metalloprotease which alters endogenous auxin levels. *needle1* displays severe reproductive defects [14].

RNA sequencing is an efficient transcriptomic technology [15]. Many genes have been identified as being involved in grain development [16, 17]. However, few studies have used large-grain mutants. Chang 7-2 is one of the maize elite inbred lines in China and has made great contributions to the cultivation of high-yield maize hybrids. *tc19* is a large-grain mutant that was selected from Chang7-2 following Co60 gamma-ray radiation. By using RNA sequencing, we analyzed the transcriptomic differences between *tc19* and Chang7-2 and identified potential genes related to grain development.

## Results

### Grain size and grain weight

To elucidate the consequence of mutations on grain size development, we performed morphological analysis using *tc19* and Chang7-2 in two locations for 2 years. We found that the length, width, thickness, and 100-kernel weight of the mature seeds of *tc19* were significantly greater than in Chang7-2 (Table 1). Grain length in *tc19* increased by 3.57%, grain width increased by 8.8%, and grain thickness increased by 3.88% compared with Chang7-2. The grain volume and 100-kernel weight of *tc19* increased by 18.75 and 16.92%, respectively. However, ear length and ear weight in *tc19* were significantly lower than in Chang7-2 (Table 1).

**Table 1:** Grains develop differently between Chang7-2 and *tc19*

Trait	Chang7-2	*tc19*	Increased percentage
Grain length (mm)	9.23 ± 0.59	9.56 ± 0.74	3.57%^b^
Grain width (mm)	7.50 ± 0.53	8.16 ± 0.81	8.80%^b^
Grain thickness (mm)	4.64 ± 0.61	4.82 ± 0.64	3.88%^b^
Grain length/width	1.23 ± 0.12	1.17 ± 0.11	−4.88%^a^
Grain volume (cm^3^)	0.32 ± 0.002	0.38 ± 0.003	18.75%^a^
100 kernel weight (g)	21.45 ± 0.72	25.08 ± 0.55	16.92%^b^
Kernel row number	14.00 ± 0.50	16.00 ± 0.80	Not Significant
Ear length (cm)	12.69 ± 1.07	7.84 ± 1.16	−38.22%^b^
Ear width (cm)	4.06 ± 0.09	4.53 ± 0.05	11.58%^b^
Ear weight (g)	93.94 ± 4.85	70.76 ± 3.38	− 24.68%^b^

^a^ *p*<0.05, ^b^ *p*<0.01

**Fig. 1:**
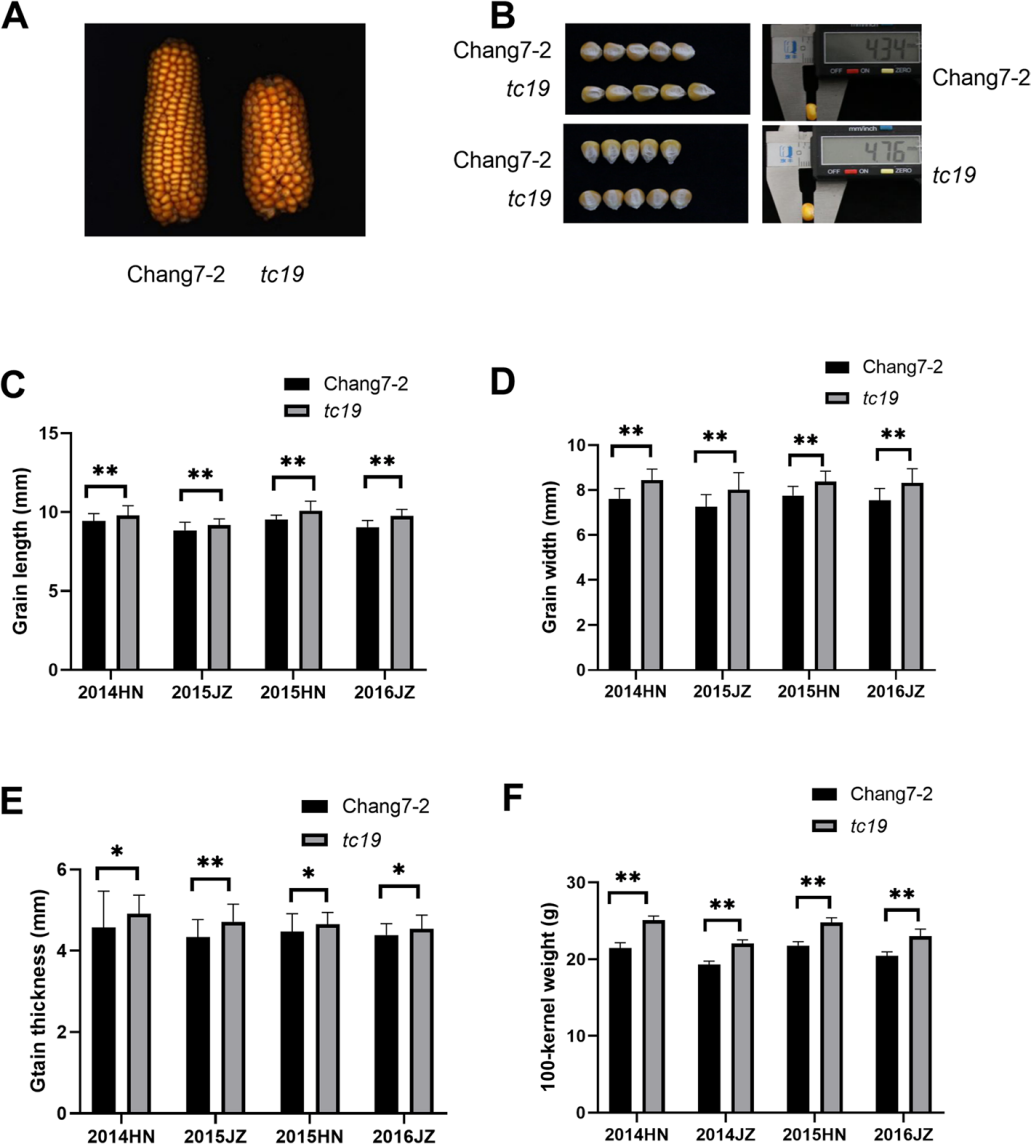
The differences in grain size between Chang7-2 and *tc19*. **A** and **B** Photographs of ears and grains of Chang7-2 and *tc19*. **C**-**F** Statistic analysis for grain size between Chang7-2 and *tc19*. ns, not significant. Values are the mean ± standard deviation. * *p* < 0.05. ** *p* < 0.01. 50 biological replicates were used for grain length, grain width, grain thickness and 100-kernel weight

Environmental factors have a great influence on plant growth and development. In this study, the grain length, grain width, grain thickness, and 100-kernel weight of Chang7-2 and *tc19* were influenced significantly by the environment. However, the grain length, grain width, grain thickness, and 100-kernel weight of *tc19* were significantly greater than those of Chang7-2 in every environment (Fig. 1), indicating that grain size is mainly controlled by genetic factors.

**Fig. 2:**
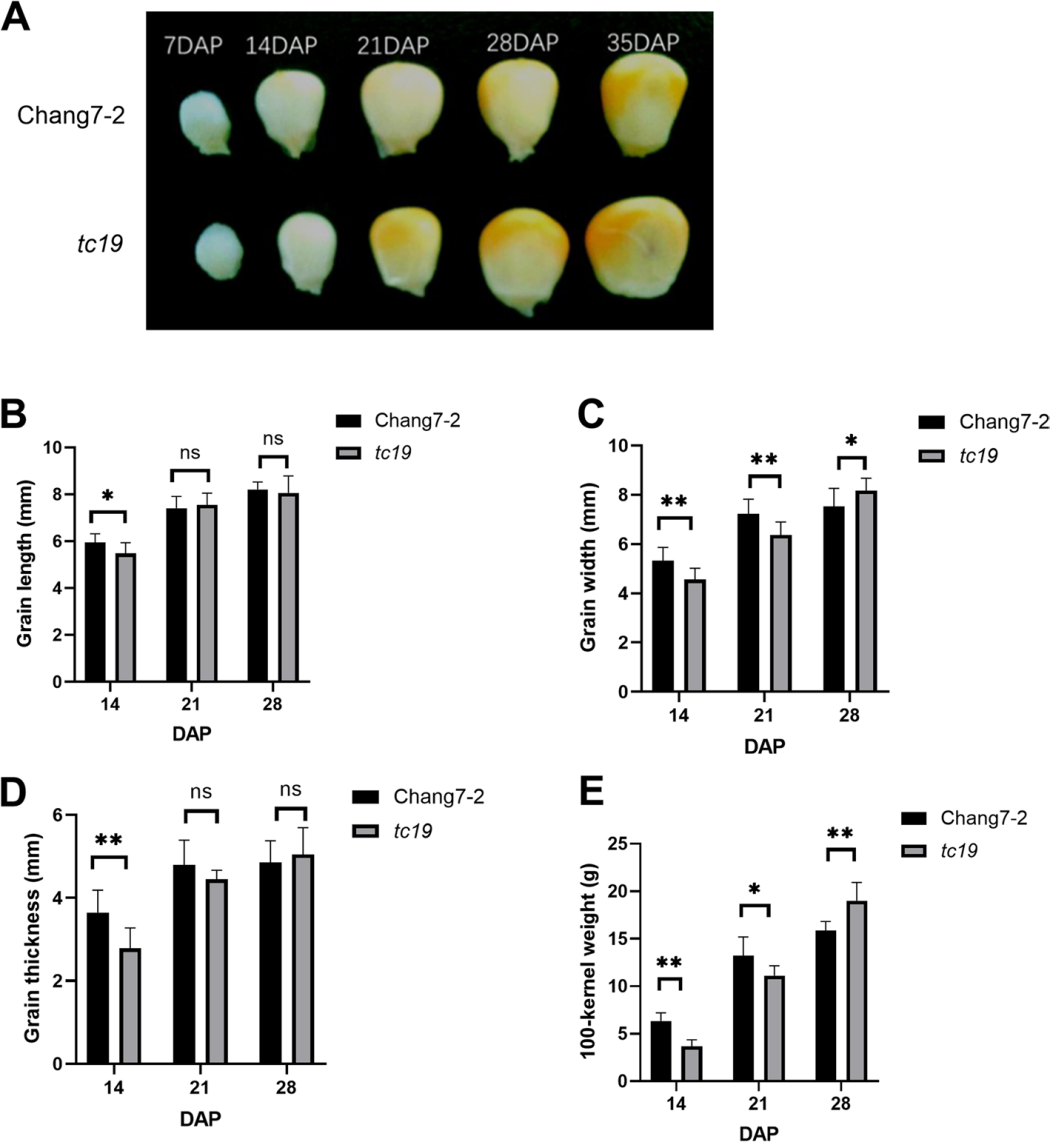
Grain development of Chang7-2 and *tc19* at different DAPs. **A** Photograph showing the difference between Chang7-2 and *tc19* at different DAPs. **B** Statistical analysis of grain width between Chang7-2 and *tc19* at different DAPs. **C** Statistical analysis of grain width between Chang7-2 and tc19 at different DAPs. **D** Statistical analysis of grain thickness of Chang7-2 and tc19. **E** Statistical analysis of 100-kernel weight of Chang7-2 and tc19. Values are the mean ± standard deviation. ns, not significant. * *p* < 0.05. ** *p* < 0.01. Three biological replicates were used for grain length, grain width, grain thickness and 100-kernel weight

Grain width changed most obviously between the mature seeds of *tc19* and Chang7-2. To determine the stage at which this difference occurred, we measured the grain width from 14 to 28 days after pollination (DAP) every 7 days. Before 21 DAP, the grain width of *tc19* was significantly smaller than that of Chang7-2. However, after 28 DAP, the grain width of *tc19* was significantly larger than that of Chang7-2. The grain width of *tc19* increased rapidly from 14 to 28 DAP, which ultimately contributed to the difference between *tc19* and Chang7-2 (Fig. 2).

### Endogenous hormones

**Fig. 3:**
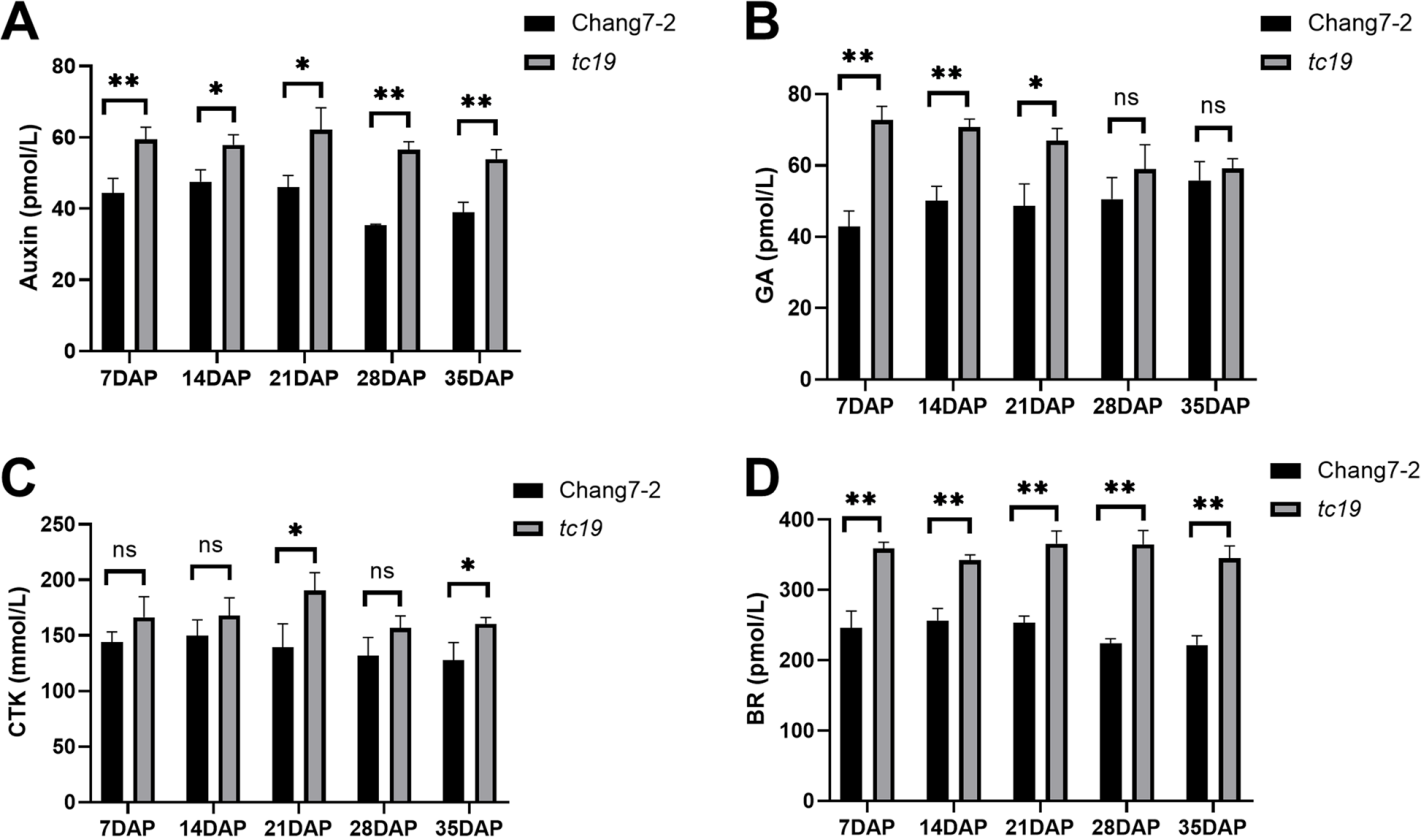
The concentration of hormones at different DAPs. **A** The concentration of auxin at different DAPs. **B** The concentration of GA at different DAPs. **C** The concentration of CTK at different DAPs. **D** The concentration of BR at different DAPs. Values are the mean ± standard deviation. ns, not significant. * *p* < 0.05. ** *p* < 0.01. Three biological replicates were used

Plant endogenous hormones, indole-3-acetic acid (Auxin), gibberellins (GAs), cytokinin (CTK) and brassinosteroids (BR), play important roles in the regulation of seed size. To elucidate the effects of hormones between *tc19* and Chang7-2, we detected the concentrations of mixed hormones at five stages after pollination (Table S1). The concentrations of auxin and BR in *tc19* were higher than those in Chang7-2 at all five stages (Fig. 3). The concentration of auxin in *tc19* peaked at 21 DAP, following which it decreased slightly at 28 and 35 DAP. The concentration of auxin in Chang7-2 also peaked at 21 DAP, reaching its lowest level at 28 DAP and then increasing slightly at 35 DAP (Fig. 3A). The concentration of GA decreased in *tc19* but increased in Chang7-2 from 7 to 35 DAP. The concentration of GA in *tc19* was significantly higher than that in Chang7-2 from 7 to 21 DAP and was no longer significant after 28 DAP (Fig. 3B). The concentration of CTK in *tc19* was higher than that in Chang7-2 at 21 DAP, but it was not significantly different at 7, 14, 28, and 35 DAP (Fig. 3C). The concentration pattern of BR was similar to that of auxin (Fig. 3D).

### Sequencing data quality assessment

First, all of the parameters for RNA quality met the library construction standards. After Illumina sequencing, we generated 20-35 M reads for the samples (Table 2). Q30 for all the samples ranged from 93.8 to 94.7%, the GC content ranged from 52.76 to 56.22% (Table S2). After alignment, more than 70% of the reads in each sample were aligned to the reference B73 genome sequence (Zm-B73-REFERENCE-NAM-5.0) (Table 2). PCA analysis successfully classified samples into different groups (Fig. S1). The pearson correlation among different groups were higher than 0.97 (Fig. S2). These results confirmed that the data were reliable.

**Table 2:** Large amounts of reads generated from samples

Sample	Total Reads	Unmapped Reads	Unique Mapped Reads	Mapping Ratio
CK-1-1	29,162,394	27.44%	71.10%	72.56%
CK-1-2	30,751,438	27.47%	71.11%	72.53%
CK-1-3	31,965,840	27.63%	70.84%	72.37%
CK-2-1	31,285,730	27.34%	71.24%	72.66%
CK-2-2	20,401,138	27.23%	71.32%	72.77%
CK-2-3	27,313,866	26.87%	71.66%	73.13%
CK-3-1	33,449,740	28.13%	70.51%	71.87%
CK-3-2	30,453,088	28.65%	70.14%	71.35%
CK-3-3	25,112,472	28.19%	70.51%	71.81%
*tc19*-1-1	29,571,446	25.57%	73.22%	74.43%
*tc19*-1-2	27,076,892	24.64%	74.14%	75.36%
*tc19*-1-3	31,937,200	25.21%	73.64%	74.79%
*tc19*-2-1	35,862,450	26.90%	71.87%	73.10%
*tc19*-2-2	31,611,148	27.07%	71.78%	72.93%
*tc19*-2-3	28,865,264	26.73%	72.12%	73.27%
*tc19*-3-1	33,666,020	27.68%	71.20%	72.32%
*tc19*-3-2	34,859,588	28.51%	70.41%	71.49%
*tc19*-3-3	34,154,116	28.30%	70.63%	71.70%

### Differential gene statistics

**Fig. 4:**
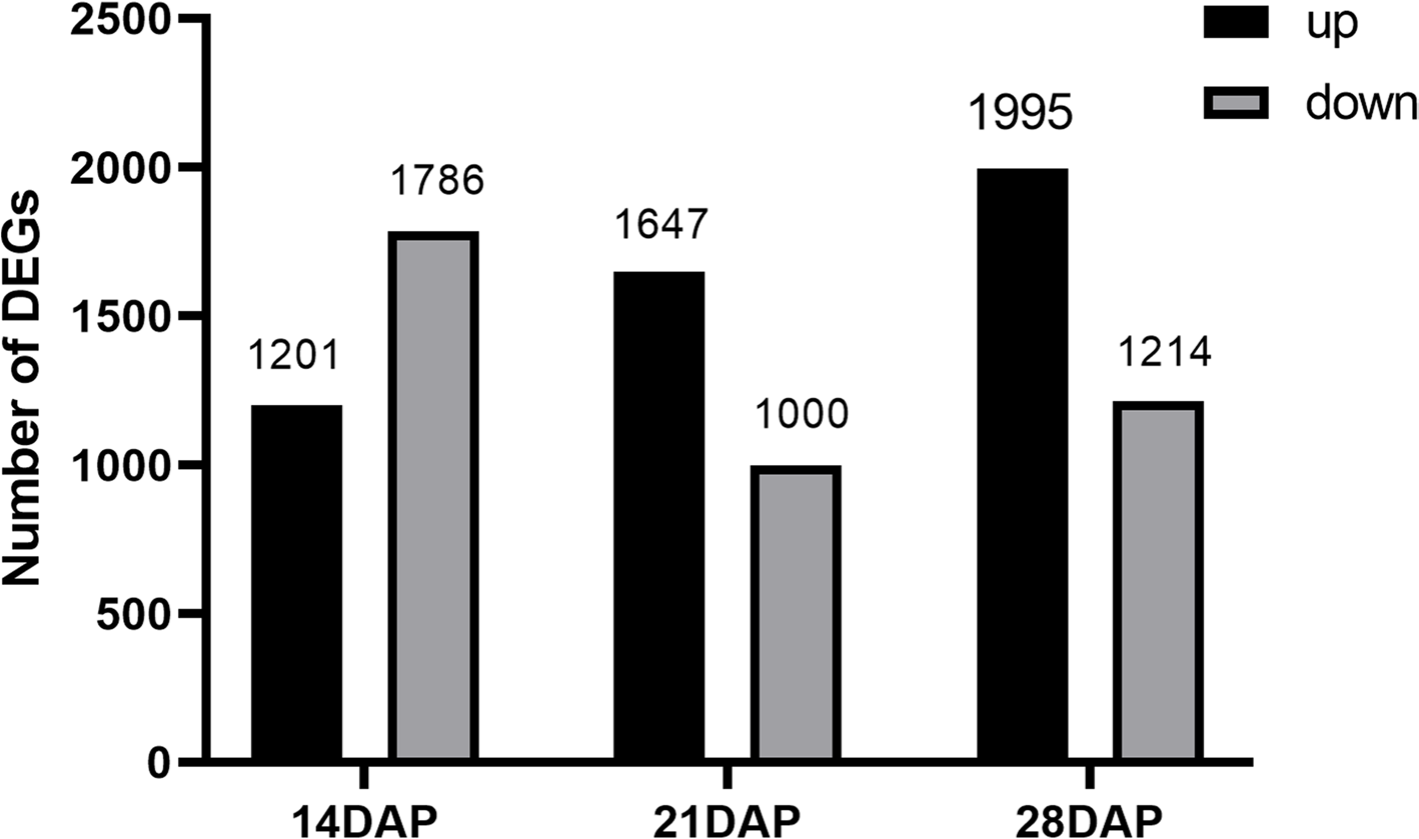
The number of differently expressed genes at 14, 21 and 28 DAP

We then screened differentially expressed genes (DEGs) between Chang7-2 and *tc19* at three stages after pollination. There were 2987, 2647, and 3209 DEGs at 14, 21, and 28 DAP, respectively. Compared with Chang7-2, 1201 genes increased and 1786 genes decreased in *tc19* at 14 DAP. A total of 1647 genes increased and 1000 genes decreased in *tc19* at 21 DAP, and 1995 genes increased and 1214 genes decreased in *tc19* at 28 DAP (Fig. 4).

### Gene ontology (GO) analysis

**Fig. 5:**
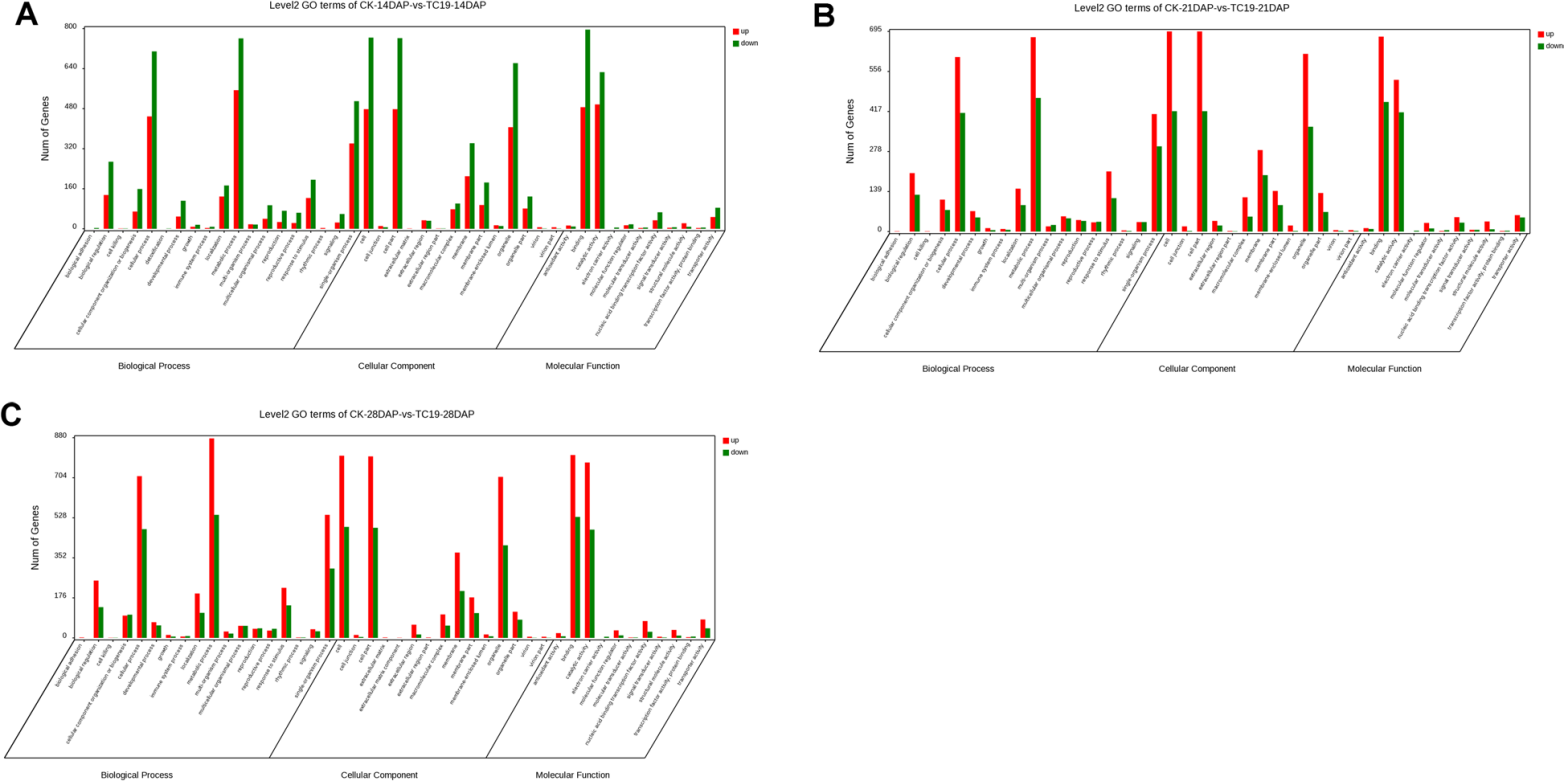
Histograms of GO classifications at different DAPs. **A** GO classifications of Chang7-2 and *tc19* at 14 DAP. **B** GO classifications of Chang7-2 and *tc19* at 21DAP. **C** GO classifications of Chang7-2 and *tc19* at 28DAP

To know the function of the DEGs, we performed GO analysis (Fig. 5). The cellular components involved at 14 DAP include lipid particle, extracellular region, nucleus, cell wall, proteasome core complex, microtubule associated complex, external encapsulating structure. The molecular functions at 14 DAP are hydrolase activity, tetrapyrrole binding, DNA binding, transcription cofactor activity. The biological processes at 14 DAP include DNA duplex unwinding, carbohydrate metabolic process, axis specification and others (Fig. 5A).

The cellular components at 21 DAP include chromatin, extracellular region, chromosomal part, cell periphery et al. The molecular function of the DEGs at 21 DAP are related to oxidoreductase activity, protein dimerization activity, oxidoreductase activity, endopeptidase inhibitor activity et al. The biological processes of the DEGs at 21 DAP include transcription, nucleic acid-templated transcription, response to heat, regulation of hydrolase activity et al. (Fig. 5B).

The cellular components of the DEGs at 28 DAP include extracellular region, cell periphery, proteasome core complex, plasma membrane et al. The molecular function of the DEGs at 28 DAP are involved in oxidoreductase activity, tetrapyrrole binding, catalytic activity, peptidase regulator activity et al. The biological processes of the DEGs at 28 DAP include peptidase activity, proteolysis, hydrolase activity, catalytic activity et al. (Fig. 5C).

### Kyoto encyclopedia of genes and genomes enrichment (KEGG) analysis

**Fig. 6:**
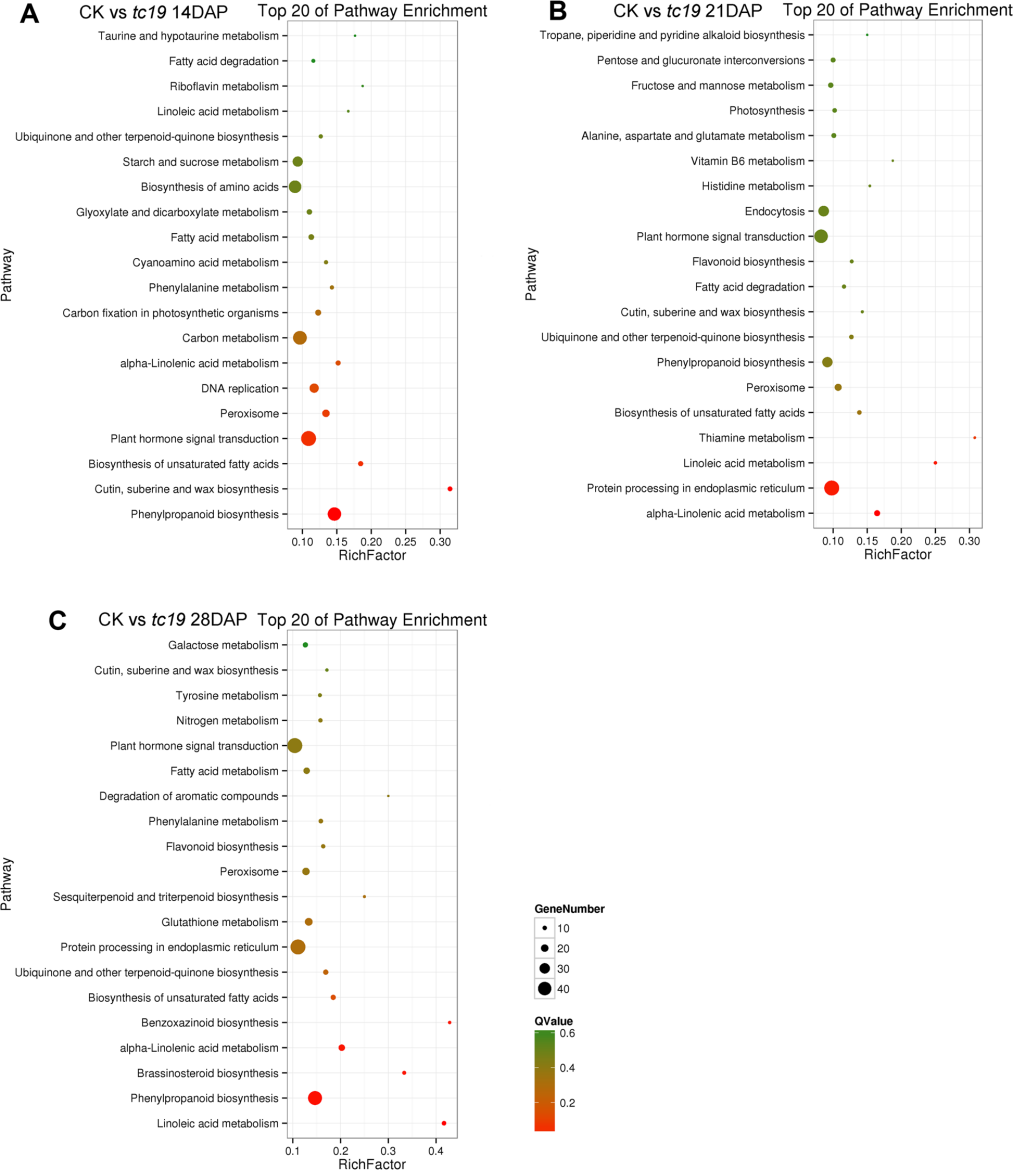
Clusters of KEGG classifications at different DAPs. **A** Clusters of KEGG classification at 14 DAP. **B** Clusters of KEGG classification at 21 DAP. **C** Clusters of KEGG classification at 28 DAP

To determine the biochemical metabolic pathways and signal transduction pathways associated with the DEGs, we performed KEGG analysis. 556, 500 and 633 DEGs at 14, 21 and 28 DAP were pathway annotated, respectively. The DEGs at 14 DAP were enriched mainly in the phenylpropane biosynthesis pathway, plant hormone signal transduction, phenylalanine metabolism, and starch sucrose metabolism pathway (Fig. 6A). The DEGs at 21 DAP were enriched mainly in endoplasmic reticulum protein processing, plant hormone signal transduction, phenylpropanoid biosynthesis, and α-linolenic acid metabolic pathways (Fig. 6B). The DEGs at 28 DAP were enriched mainly in phenylpropanoid biosynthesis, plant hormone signal transduction, brassinosteroid synthesis, and α-linolenic acid metabolism (Fig. 6C). Above all, the DEGs in the hormone signal transduction pathway were significantly enriched. This indicated that the signal transduction pathway may play an important role in seed development.

### Genes enriched in hormone signal transduction

**Fig. 7:**
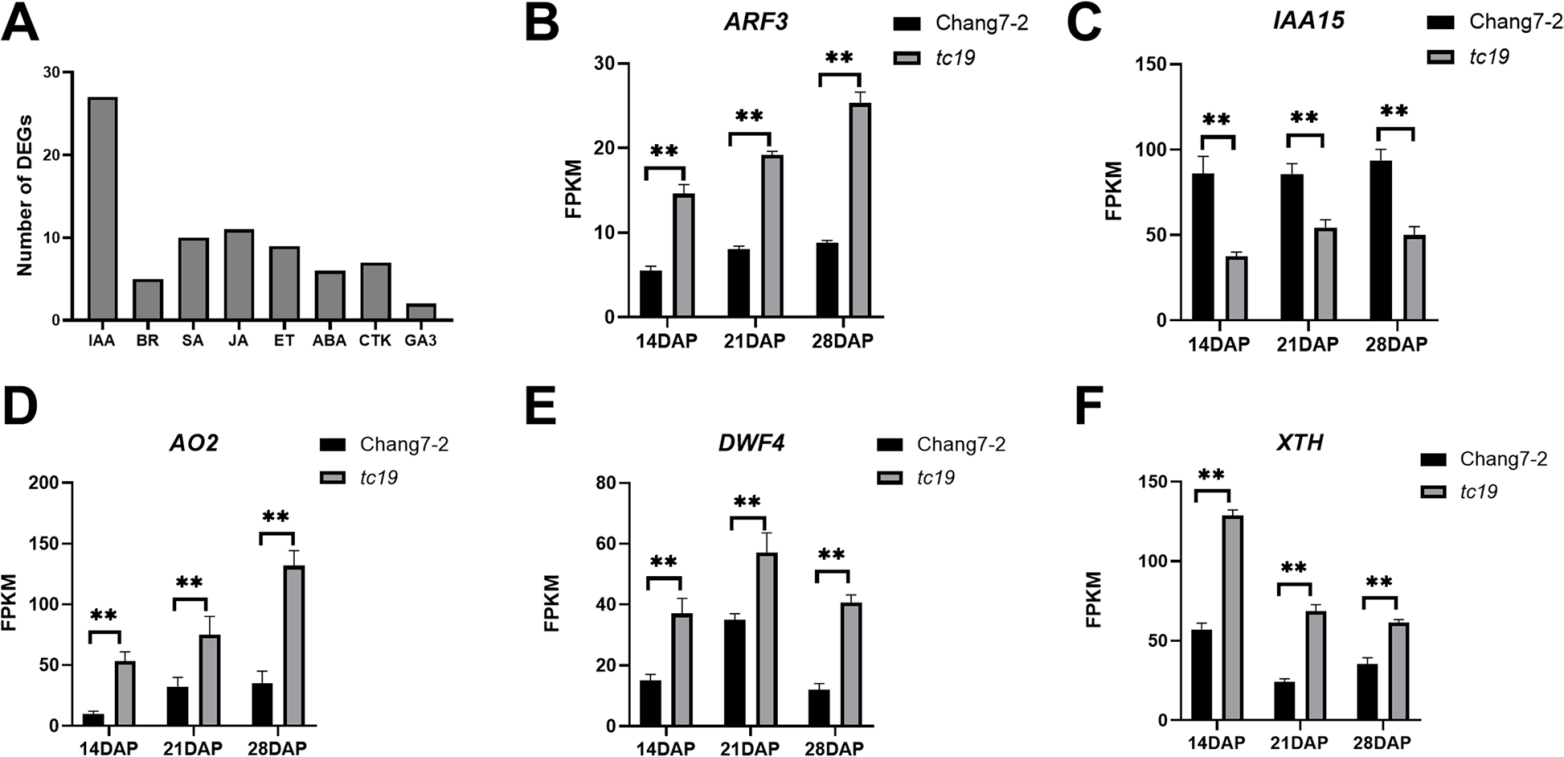
The expression of several hormone-related genes. **A** The number of DEGs related to hormones. **B** The expression of ARF3. **C** The expression of IAA5. **D** The expression of AO2. **E** The expression of DWF4. **F** The expression of XTH. Values are the mean ± standard deviation. ns, not significant. * *p* < 0.05. ** *p* < 0.01. Three biological replicates were used

We found a total of 77 DEGs related to the hormone signal transduction pathway (Fig. 7A). Among them, 27 genes were involved in the IAA signal transduction pathway; 5 genes were involved in the BR signal transduction pathway; 7 genes were involved in the CTK signal transduction pathway; 2 genes were involved in the GA signal transduction pathway; 6 genes were involved the abscisic acid (ABA) signal transduction pathway; 9 genes were involved in the ethylene (ET) signal transduction pathway; 11 genes were involved in the jasmonic acid (JA) signal transduction pathway; and 10 genes were involved in the SA signal transduction pathway.

We detected 27 DEGs involved in the IAA signal transduction pathway. *ARF3* (*Zm00001d012731*) and *IAA15* (*Zm00001d039624*) showed high expression levels. The expression level of *ARF3* in *tc19* was higher than that in Chang7-2. From 14 to 28 DAP, the expression of *ARF3* was significantly increased in *tc19*, whereas it increased only slightly in Chang7-2 (Fig. 7B). The expression of *IAA15* in Chang7-2 was higher than that of *tc19* (Fig. 7C). *AO2* (*Zm00001d034388*) in *tc19* was higher than that in Chang7-2 (Fig. 7D). Endogenous hormone analysis showed that the BR concentrations of Chang7-2 and *tc19* differed significantly. Analysis of the BR biosynthesis pathway indicated that *DWF4* (*ZM00001d003349*) and *XTH* (*Zm00001d014617*) were highly expressed in *tc19* than in Chang7-2 (Fig. 7E and F).

## Discussion

In this study, we used the *tc19* maize mutant, which had been screened after Co60-γ-ray irradiation and had been self-pollinated for multiple generations on the background of a maize inbred line Chang7-2. the grain length, grain width, grain thickness, and 100-kernel weight of *tc19* were significantly increased, whereas the ear length and grain weight were reduced, comparing with Chang7-2. Kernel number per grain, 100-kernel weight, and ear number are critical components of maize yield. The phenomenon of improved grain weight with reduced yield has been observed previously [18]. Some quantitative trait loci (QTLs) have been found to affect the balance between the kernel and ear [19, 20]. Thus, the relationship between kernel, ear per plant, and field conditions should be carefully considered in plant breeding.

The grain type and grain weight of maize seeds are controlled by both genetic and environmental factors, such as temperature, moisture, disease, and insect pests. To explore the difference between the grain development of Chang7-2 and *tc19*, we analyzed grain length, grain width, grain thickness, and 100-kernel weight under multiple different environmental conditions. The results showed that environments have an effect on grain size. However, the grain size and weight of *tc19* under each environmental condition were always higher than that in Chang7-2, indicating that grain development is mainly genetically controlled. This is consistent with previous studies [21].

In this study, grain width was the main contributor to the difference in grain size between Chang7-2 and *tc19*. The grain width increased fastest in *tc19* from 14 to 28 DAP, at which stage it exceeded Chang7-2, indicating that the stage of 14 to 28 DAP is an important period for grain enlargement. Some studies showed that this period is the grain-filling stage which is critical for grain width development [22].

Plant hormones are one of the most important factors affecting the growth and development of grains [23, 24]. Cytokinin and brassinolide play a vital role in regulating seed size, auxin, ABA, and gibberellin have regulatory effects on seed development to a certain extent [25]. Through transcriptomics analysis, we found that the signal transduction pathway has a notable influence on grain size. Five genes had exhibited high expression levels in this study, including *ARF3*, *IAA15*, *AO2*, *DWF4*, and *XTH*. *ARF3*, *IAA15*. *AO2* are related to the IAA biosynthesis or signal transduction pathway, and *DWF4* and *XTH* are related to the BR biosynthesis or signal transduction pathway [26].

The Aux/IAA protein, as a type of transcription inhibitor, has been proven to play an important role in the auxin signal transduction pathway. Generally, the auxin response requires the degradation of the Aux/IAA inhibitor. After that, the ARF transcription factor can be released to regulate the target genes. *ARF3* belongs to the ARF family [27] while *IAA15* is a member of the AUX/IAA family [28]. In Chang7-2, the expression of *ARF3* is very low while *IAA15* is high expressed. However, in *tc19*, the expression of *ARF3* is much higher. Indicating that *ARF* is released in *tc19*, which is consistent with the previous study. *AO2* encodes 3-indole acetaldehyde oxidase, a key enzyme in the indolepyruvate pathway [29]. In this study, the expression of the *AO2* gene in *tc19* was higher than that in Chang7-2, which is consistent with the endogenous IAA measurement results.

*DWF4* encodes sterol C-22α hydroxylase, which acts as the rate-limiting link in the process of BR biosynthesis. A high expression of *DWF4* increases the BR content in grains [30]. In this study, the expression level of *DWF4* in *tc19* was higher than that in Chang7-2, which is consistent with the higher BR content in *tc19*. *XTH* encodes xyloglucan endotransglycosidase/hydrolase, which is a cell wall relaxase and a key enzyme in plant cell wall remodeling. Studies have shown that *XTHs* play roles in cell volume growth, and their expression is induced by BR [31]. The higher expression level of *XTH* is consistent with the higher BR content in *tc19*.

In addition to the genes related to auxin and brassinolide, some genes related to other hormones were differentially expressed between Chang7-2 and *tc19*. We propose that auxin and brassinolide contribute significantly to the enlarged size of the tc19 grains. The molecular regulatory mechanism of plant seed size is complex, and many genes are waiting to be identified in this process. To study the regulatory mechanisms of seed size, genes needs to be cloned and functionally characterized.

## Conclusion

The grain width and 100-kernel weight in *tc19* are greater than that in Chang7-2. The concentrations of IAA, BR, GA and CTK were higher in *tc19* than in Chang7-2. 2987, 2647, and 3209 differentially expressed genes (DEGs) identified between *tc19* and Chang7-2 at 14, 21, and 28 DAP, respectively. GO and KEGG analysis found that 77 DEGs are enriched in the plant hormone signal transduction pathway. The expression of *ARF3*, *IAA15*, *AO2*, *DWF4* and *XTH* may explain the grain developmental difference between *tc19* and Chang7-2.

## Methods

### Plant growth and phenotyping

The seeds of Chang7-2 were obtained from the maize center of Qingdao Agricultural University. *tc19* was originally generated after Co60-γ radiation on Chang7-2 background in the Song lab in Qingdao Agricultural University. The permission of seeds collection has been obtained. Chang7-2 and *tc19* were sown in Sanya (SY, 18°30′N, 108°47′E) in 2014 and 2015, and Jiaozhou (JZ, 36°04′N, 120°18′E) in 2015 and 2016. Single seeds were sown with a 3-m row length, 0.6-m row spacing, and 0.2-m plant spacing, with 10 rows per material under normal field management practices. All plants were self-pollinated. Cobs were taken at 7 days, 14 days, 21 days, 28 days, and 35 days after pollination. Grains were isolated from the center of the cobs at the same growth stage. For each treatment, three cobs were selected, and when the maize was mature, they were single-ear harvested and dried naturally to a water content of about 13%. Afterward, at least three ears were selected for measurement. Grains at the same growth stage and of the same shape were selected for measurement of kernel length, width, thickness, and 100-kernel weight. The data were analyzed using Excel 2016 and Graphpad Prism 8. We declare that all the collections of plant and seed specimens related to this study were performed in accordance with the relevant guidelines and regulations by Ministry of Agriculture of the People’s Republic of China.

### Determination of endogenous hormone content

The maize inbred lines Chang7-2 and *tc19* were sown in the Modern Agricultural Science and Technology Demonstration Park of Qingdao Agricultural University in 2016. After tasseling, they were all self-pollinated. Cobs were sampled at 7, 14, 21, 28, and 35 DAP. Grains were isolated from the center of the cobs at the same growth stage, and more than three cobs were sampled for each treatment. Hormones were tested by using Auxin Elisa Kit, GA ELISA Kit, BR Elisa Kit and BR Elisa Kit.

Samples of 0.2 to 0.5 g were rinsed in ice-cold PBS (0.05 mol/L Tris-HCl, pH = 7.4), wiped dry with filter paper, weighed accurately, and placed into a 5 ml homogenization tube. Four times the volume of homogenization medium was added to the tube at the ratio of weight (mg): volume (ml) 1:4, and the tissue was cut as soon as possible using small ophthalmic scissors in an ice water bath. A masher was used for grinding the tissue at 10000 to 15,000 r/min. A small amount of tissue homogenate was used for smearing, broken cells were observed under a microscope. The sample was then centrifuged at 4000 r/min for 10 to 15 min, and the supernatant was used for determination.

First, set the blank wells and sample wells a plate. Forty microliters of sample diluent were added to each well, following which 10 μl of the sample solution was added. Fifty microliters of conjugate reagent was added to each well, except for the blank wells. The plate was sealed with closure plate membrane and incubated for 30 min at 37 °C. Then, the liquid was discarded. Each well was filled with the washing solution and incubated for 30 s, then, the washing solution was discarded. This step was repeated 5 times. Fifty microliters of chromogen solution A and chromogen solution B were added to the wells, the plate was gently mixed, incubated for 15 min at 37 °C in the dark. Then, 50 μl of stop solution was added to each well. Finally, the OD value at 450 nm wavelength of each well was measured using a microtiter plate reader.

Taking the concentration of the standard substance as the ordinate (Y) and the OD value of our samples as the abscissa (X), we calculated the polynomial quadratic regression equation of the standard curve. The quadratic regression equation of each hormone was as follows:

$${\displaystyle \begin{array}{l}\mathrm{Gibberellin}\ \left(\mathrm{GA}\right):\mathrm{Y}=0.4303+34.5196\mathrm{X};\\ {}\mathrm{Auxin}\ \left(\mathrm{IAA}\right):\mathrm{Y}=-1.6192+32.3868\mathrm{X};\\ {}\mathrm{Cytokinin}\ \left(\mathrm{CTK}\right):\mathrm{Y}=1.1722+21.0967\mathrm{X};\\ {}\mathrm{Brassinolide}\ \left(\mathrm{BR}\right):\mathrm{Y}=6.8315+83.9345\mathrm{X}.\end{array}}$$

### RNA extraction

Total RNA was extracted using Trizol according to the standard protocol. The grains were ground into powder in liquid nitrogen and placed in a 2 ml Eppendorf tube. One thousand five hundred microliters of the extraction reagent TRNzol-A+ were added, vortexed thoroughly and incubated at room temperature for 30 min. The sample was then centrifuged at 12000 rpm for 10 min, the supernatant was transferred to a new RNase-free 2 ml Eppendorf tube. Three hundred milliliters of chloroform/isoamyl alcohol (24:1) was added and mixed, incubated at room temperature for 15 min. The sample was then centrifuged at 12000 rpm at 4 °C for 15 min, and then 500 μl of the supernatant was transferred to a new RNase-free centrifuge tube. Five hundred microliters isopropanol (pre-cooled at − 20 °C) was added to the tube, mixed well and incubated at room temperature for 15 min. After centrifugated at 12000 rpm for 10 min at 4 °C, the supernatant was discarded. One milliliter of pre-cooled 75% ethanol was added to the centrifuge tube, shaken gently and centrifuged at 4 °C and 12,000 rpm for 3 min. When the ethanol had evaporated, 40 μl of RNase-free water was added and mixed by pipetting. RNA quality was assessed on an Agilent 2100 Bioanalyzer using RNA 6000 Nano kit (Agilent Technologies, Palo Alto, CA, USA) and checked using RNase free agarose gel electrophoresis.

### Library construction and sequencing

The enriched mRNA was fragmented into short fragments using fragmentation buffer and reversly transcribed into cDNA by using NEBNext Ultra RNA Library Prep Kit for Illumina (NEB #7530, New England Biolabs, Ipswich, MA, USA). The purified double-stranded cDNA fragments were end repaired, base A added, and ligated to Illumina sequencing adapters. The ligation reaction was purified with the AMPure XP Beads(1.0X). The Ligated fragments were subjected to size selection by agarose gel electrophoresis and polymerase chain reaction (PCR) amplified. The resulting cDNA library was sequenced using Illumina HiSeqTM 2500 by Gene Denovo Biotechnology Co. (Guangzhou, China).

### Alignment with reference genome

The sequencing data analysis was performed by Gene Denovo Biotechnology Co. (Guangzhou, China). The raw image data measured by the Illumina HiSeqTM 2500 was converted into sequence data by using the Base Calling. Reads with more than 10% of unknown nucleotides and low-quality reads containing more than 50% of low quality (Q-value≤20) bases were removed. The clean reads were aligned and assembled to the maize B73 reference genome (Zm-B73-REFERENCE-NAM-5.0) by using TopHat2 and Cufflinks, respectively. The genome data was downloaded from Ensembl Plants (http://plants.ensembl.org/Zea_mays/Info/Index).

#### Gene expression analysis

The mapped reads of each sample were reconstructed into transcripts by using StringTie v1.3.1. For each transcription region, a FPKM (fragment per kilobase of transcript per million mapped reads) value was calculated to quantify its expression abundance and variations, using RSEM software. Correlation analysis was performed by R. Principal component analysis (PCA) was performed with R package gmodels (http://www.rproject.org/). RNAs differential expression analysis was performed by edgeR between two samples. The genes/transcripts with the parameter of false discovery rate (FDR) below 0.05 and absolute fold change≥2 were considered differentially expressed genes/transcripts.

#### GO enrichment analysis

The DEGs were mapped to each term in the GO database (http://www.geneontology.org/) and the number of genes in each term with GO functions and gene item statistics were calculated. A hypergeometric test was performed to determine the GO entries that were significantly enriched in the DEGs.

#### Pathway enrichment analysis

Kyoto Encyclopedia of Genes and Genomes (KEGG) is the main public database on pathways. Pathway-significant enrichment analysis uses KEGG pathways as the unit and applies hypergeometric tests to determine pathways that are significantly enriched in DEGs compared with the entire genome background.

## Supplementary Information

**Additional file 1. Figure S1. **The samples classified into different groups based on PCA analysis.

**Additional file 2. Figure S2. **High correlation between each of the two samples.

**Additional file 3: Table S1.** Different hormones concentrations between Chang7-2 and *tc19*.

**Additional file 4: Table S2.** High quality of RNA samples.

## Data Availability

The raw sequence data are available in the NCBI Sequence Read Archive (SRA) repository. The accession number is PRJNA724904, the website link is https://dataview.ncbi.nlm.nih.gov/object/PRJNA724904. All data supporting the conclusions of this article are included in the article and its additional files.

## Abbreviations

DAP: Days after pollination
IAA: Indole-3-acetic acid
BR: Brassinosteroids
GAs: Gibberellins
CTK: Cytokinin
DEGs: Differentially expressed genes
ARF3: AUXIN RESPONSE FACTOR 3
IAA15: Auxin-responsive protein IAA15
AO2: Aldehyde oxidase 2
DWF4: Dwarf4
XTH: Xyloglucan endotransglycosidase/hydrolase
GO: Gene Ontology
KEGG: Kyoto Encyclopedia of Genes and Genomes enrichment analysis
