# Supplementary figures and images for: Transcriptomic analysis of the maize inbred line Chang7-2 and a large-grain mutant tc19
Source: BMC Genomics. 2022 Jan 4;23:4. doi: 10.1186/s12864-021-08230-9 (PMC8725412; doi:10.1186/s12864-021-08230-9)

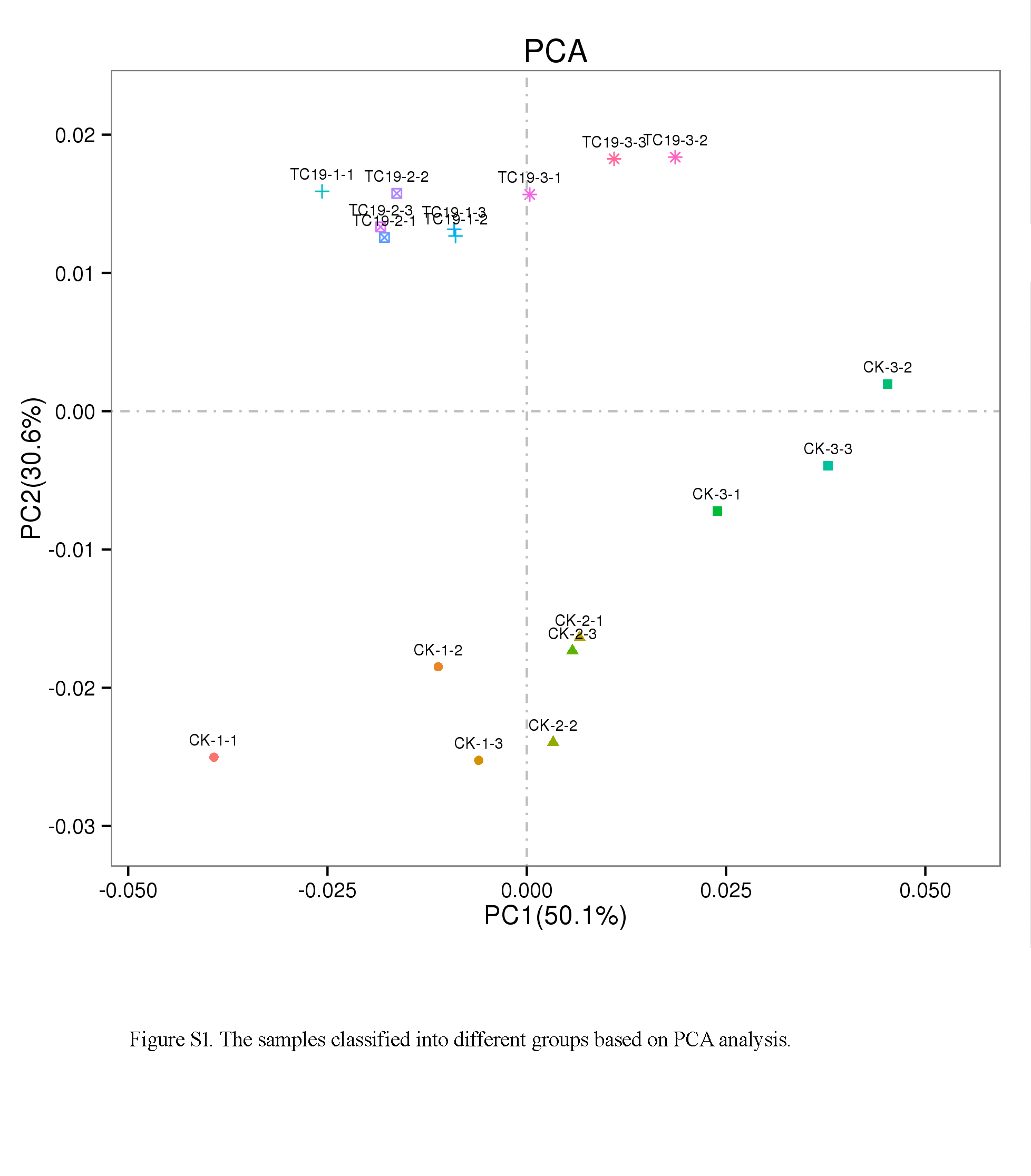

Supplement: Supplementary file 1 — Additional file 1. Figure S1. The samples classified into different groups based on PCA analysis. [file 12864_2021_8230_MOESM1_ESM.png]

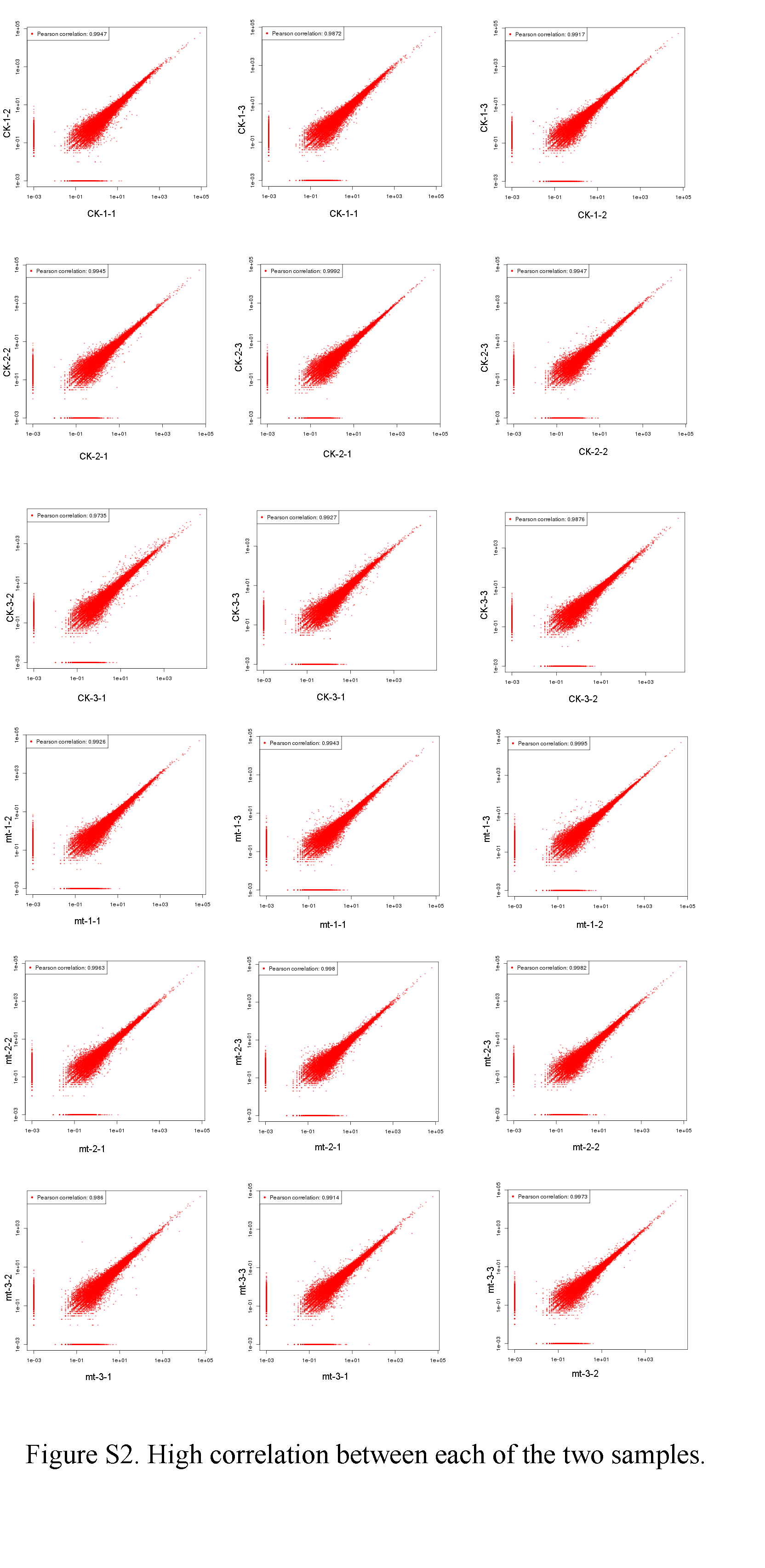

Supplement: Supplementary file 2 — Additional file 2. Figure S2. High correlation between each of the two samples. [file 12864_2021_8230_MOESM2_ESM.png]
