# Supplementary material for: Transcriptomic analysis of the maize inbred line Chang7-2 and a large-grain mutant tc19
Source: BMC Genomics. 2022 Jan 4;23:4. doi: 10.1186/s12864-021-08230-9 (PMC8725412; doi:10.1186/s12864-021-08230-9)
Supplement: Supplementary file 3 — Additional file 3: Table S1. Different hormones concentrations between Chang7-2 and tc19. [file 12864_2021_8230_MOESM3_ESM.docx]

| **Hormomes** | **#** | **7DAP** | **14DAP** | | **21DAP** | **28DAP** | **35DAP** |
| --- | --- | --- | --- | --- | --- | --- | --- |
| **IAA** | 1 | 44.42±4.05 | 47.56±3.34 | 46.04±3.28 | | 35.3±0.32 | 38.86±2.93 |
| (pmol/L) | 2 | 59.43±3.40** | 57.92±2.75* | 62.18±6.11* | | 56.57±2.20** | 53.92±2.65** |
| **GA3** | 1 | 42.88±4.34 | 50.19±4.0 | 48.64±6.20 | | 50.54±6.09 | 55.83±5.27 |
| (pmo/L) | 2 | 72.69±3.88** | 70.79±2.25** | 66.99±3.37* | | 59.05±6.81 | 59.17±2.76 |
| **CTK** | 1 | 144.01±9.21 | 149.78±14.34 | 139.48±20.97 | | 132.03±16.24 | 127.63±15.94 |
| (nmol/L) | 2 | 166.34±18.60 | 168.07±15.74 | 190.4±16.25* | | 156.53±11.17 | 160.57±5.59* |
| **BR** | 1 | 246.26±23.58 | 256.44±16.90 | 253.24±9.73 | | 224.14±6.43 | 221.52±13.15 |
| pmol/L | 2 | 358.89±9.03** | 342.30±7.49** | 365.87±18.05** | | 364.71±19.73** | 345.21±17.42** |

Table S1 Different hormones concentrations between Chang7-2 and *tc19*.
