# Supplementary material for: Transcriptomic analysis of the maize inbred line Chang7-2 and a large-grain mutant tc19
Source: BMC Genomics. 2022 Jan 4;23:4. doi: 10.1186/s12864-021-08230-9 (PMC8725412; doi:10.1186/s12864-021-08230-9)
Supplement: Supplementary file 4 — Additional file 4: Table S2. High quality of RNA samples. [file 12864_2021_8230_MOESM4_ESM.docx]

Table S2 High quality of RNA samples

| Sample | HQ-Clean Data(bp) | Q20(%) | Q30(%) | N(%) | GC(%) |
| --- | --- | --- | --- | --- | --- |
| CK-1-1 | 4427537071 | 97.86% | 94.5% | 97812(0%) | 53.49% |
| CK-1-2 | 4683089244 | 97.84% | 94.4% | 103843(0%) | 53.24% |
| CK-1-3 | 4924820149 | 97.64% | 94.0% | 109265(0%) | 52.76% |
| CK-2-1 | 4763029229 | 97.56% | 93.8% | 102979(0%) | 53.84% |
| CK-2-2 | 4625314753 | 97.90% | 94.5% | 102487(0%) | 54.08% |
| CK-2-3 | 4159181658 | 97.87% | 94.5% | 920480(0%) | 53.97% |
| CK-3-1 | 5087109714 | 97.84% | 94.4% | 114323(0%) | 54.73% |
| CK-3-2 | 4653376880 | 97.95% | 94.7% | 102824(0%) | 54.87% |
| CK-3-3 | 3861238797 | 97.85% | 94.4% | 84926(0%) | 54.51% |
| TC19-1-1 | 4502881483 | 97.87% | 94.5% | 100983(0%) | 54.94% |
| TC19-1-2 | 4145096563 | 97.84% | 94.4% | 92128(0%) | 54.09% |
| TC19-1-3 | 4880224359 | 97.77% | 94.3% | 108453(0%) | 54.09% |
| TC19-2-1 | 5421131617 | 97.80% | 94.3% | 118968(0%) | 55.22% |
| TC19-2-2 | 4767571161 | 97.97% | 94.7% | 107961(0%) | 55.92% |
| TC19-2-3 | 4362261051 | 97.85% | 94.5% | 973911(0%) | 55.16% |
| TC19-3-1 | 5108940024 | 97.90% | 94.6% | 114595(0%) | 55.70% |
| TC19-3-2 | 5278087821 | 97.74% | 94.3% | 118153(0%) | 55.77% |
| TC19-3-3 | 5172679917 | 97.90% | 94.5% | 114367(0%) | 56.22% |

Q20, a quality score of 20, a 1% chance of error and 99% confidence

Q30, a quality score of 30, a 0.1% chance of error and 99.9% confidence

N%, the ratio of unread bases in the total base number
